# RRM domain of ALS/FTD-causing FUS characteristic of irreversible unfolding spontaneously self-assembles into amyloid fibrils

**DOI:** 10.1038/s41598-017-01281-7

**Published:** 2017-04-21

**Authors:** Yimei Lu, Liangzhong Lim, Jianxing Song

**Affiliations:** Department of Biological Sciences, Faculty of Science, National University of Singapore, 10 Kent Ridge Crescent, 119260 Singapore, Singapore

## Abstract

526-residue FUS functions to self-assemble into reversible droplets/hydrogels, which could be further solidified into pathological fibrils. FUS is intrinsically prone to aggregation, composed of N-terminal low-sequence complexity (LC); RNA-recognition motif (RRM) and C-terminal LC domains. Intriguingly, previous *in vivo* studies revealed that its RRM is required for manifesting FUS cytotoxicity but the underlying mechanism remains unknown. Here, we characterized solution conformations of FUS and its five differentially dissected fragments, followed by detailed investigations on thermal unfolding, NMR dynamics and self-assembly of RRM. The results decipher: (1) the N- and C-terminal LC domains are intrinsically disordered, while RRM is folded. Intriguingly, well-dispersed HSQC peaks of RRM disappear in the full-length FUS, reminiscent of the previous observation on TDP-43. (2) FUS RRM is characteristic of irreversible unfolding. “Model-free” analysis of NMR relaxation data decodes that RRM has high ps-ns conformational dynamics even over some residues within secondary structure regions. (3) RRM spontaneously self-assembles into amyloid fibrils. Therefore, in addition to the well-established prion-like region, FUS RRM is also prone to self-assembly to form amyloid fibrils. Taken together, FUS RRM appears to play a crucial role in exaggerating the physiological/reversible self-assembly into pathological/irreversible fibrillization, thus contributing to manifestation of FUS cytotoxicity.

## Introduction

Fused in Sarcoma/Translocated in Sarcoma (FUS) consisting 526 residues is encoded by a gene which was first identified as a fusion oncogene in human liposarcomas^1, 2^. The FUS protein belongs to the FET protein family, which also includes Ewing RNA binding protein (EWS), and TATA-binding protein associated factor (encoded by TAF15)^3, 4^. Although the precise physiological functions of FUS remain to be fully elucidated, growing evidence suggests that FUS is involved in various cellular processes, including cell proliferation, DNA repair, transcription regulation, and multiple levels of RNA and microRNA processing^5–7^. On the other hand, FUS is extensively involved in the pathology of neurodegenerative diseases. FUS aggregation has been observed in amyotrophic lateral sclerosis (ALS), frontotemporal dementia (FTD), the polyglutamine diseases which include Huntington disease, spinocerebellar ataxia, and dentatorubropallidoluysian atrophy^3–10^. In addition, genetic variants in the FUS gene have been identified as causative or risk factors for ALS, essential tremor and rare forms of FTLD^11–15^. These findings suggest that FUS might have a general role in neurodegenerative diseases.

**Figure 1:**
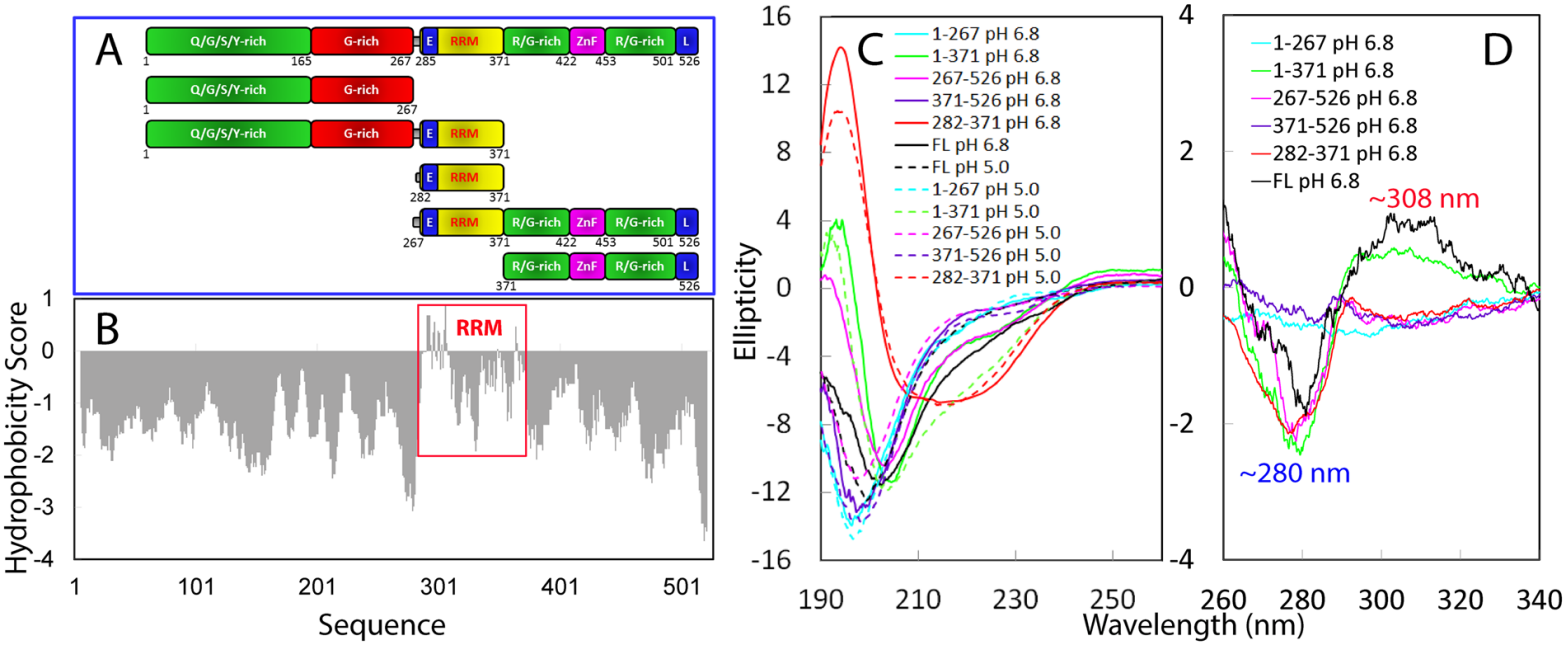
Domain organization and dissection of FUS. (**A**) FUS protein and its five differentially-dissected fragments studied here. The 526-residue FUS contains: (1) N-terminal low-sequence complexity (LC) region (1–267) including a QGSY-rich prion-like domain (1–165) and a G-rich region (166–267); RNA-recognition motif (RRM: 282–371); and C-terminal LC domain (371–526) including a RGG repeat region and a highly conserved nonclassical nuclear localization signal (L). (**B**) Kyte & Doolittle hydrophobic scale of FUS. The red box is used to indicate the RRM regions with positive scales. (**C**) Far-UV CD spectra of the full-length FUS and its five dissected fragments collected in 1-mm curvet at 25 °C at a protein concentration of 40 µM in 1 mM phosphate buffers at pH 5.0 and pH 6.8 respectively. (**D**) Near-UV CD spectra of the full-length FUS and its five dissected fragments collected in 10-mm curvet at 25 °C at a protein concentration of 40 µM in 1 mM phosphate buffers at pH 6.8.

FUS is a multi-domain protein intrinsically prone to aggregation^5–10^, which is composed of an N-terminal low-sequence complexity (LC) domain (1–267) including a QGSY-rich prion-like region (1–165) and a G-rich region (166–267); an RNA-recognition motif (RRM: 285–371) capable of binding a large array of RNA and DNA^1, 16, 17^; and C-terminal LC domain (371–526) including a RGG repeat region and a highly conserved nonclassical nuclear localization signal (Fig. 1A). RRM is one of the most abundant protein domains in eukaryotes, carrying the conserved RNP1 and RNP2 sequence stretches^18^. Most heterogeneous nuclear ribonucleoproteins (hnRNP) contain one or several RRM domains that mediate the direct interaction with nucleic acids to control both RNA processing and gene expression^19^. Noticeably, despite a large sequence variation from other RRMs, the RRM domain of FUS has been determined by NMR spectroscopy to adopt the same overall fold as other RRMs, which consists of a four-stranded β-sheet and two perpendicular α-helices. Nevertheless, the FUS RRM domain does own a unique, extra-long, and positively-charged “KK” loop essential for binding nucleic acids^17^. Very amazingly, previous *in vivo* studies revealed that RRM is required for manifesting FUS cytotoxicity but its underlying mechanism remains largely elusive^20^.

Previously, as facilitated by our discovery that unlike the well-folded proteins following the “Salting-in” rule that protein solubility increases upon adding salts over the range of low salt concentrations (usually <300–500 mM), “insoluble” proteins could only be solubilized in aqueous solution with minimized salt concentrations^21, 22^, we have successfully studied the ALS-causing and aggregation-prone TDP-43 N-terminal and C-terminal prion-like domains^23, 24^. Here, by the same approach, we characterized conformations of the full-length FUS and its five dissected domains (Fig. 1A), all of which except for the isolated RRM domain are also highly prone to aggregation. We found that out of three FUS domains, only RRM is folded while the N- and C-terminal LC domains are all intrinsically disordered. Unexpectedly, in the context of the full-length FUS protein, well-dispersed NMR HSQC peaks of the RRM domain became disappeared. To understand the underlying mechanism, we conducted further investigations on both thermodynamic and conformational stability, as well as self-assembly of the FUS RRM domain by CD, fluorescence, NMR spectroscopy and EM imaging. The results decode that the FUS RRM domain characteristic of irreversible unfolding has a large portion of the residues which undergo ps-ns conformational flexibility. Remarkably, the FUS RRM domain spontaneously self-assembles into amyloid fibrils. Therefore, our study provides key biophysical insights into the role of RRM in solidifying the FUS self-assembly, which may thus rationalize its essentiality in manifesting FUS cytotoxicity (20).

## Results

### N- and C-terminal LC domains are intrinsically disordered but RRM is folded

To have an overall view of the sequence property of FUS, we calculated its hydrophobicity^25^ and interestingly, all residues have negative score, except for a small portion of residues within the RRM domain (Fig. 1B). We first dissected FUS into three domains, namely the N-terminal LC (1–267); RRM (282–371) and C-terminal LC (371–526) domains (Fig. 1A), and subsequently cloned DNA fragments encoding them into the expression vector. While the RRM domain was highly soluble in supernatant and thus purified under native condition, the full-length FUS and two LC domains were found only in inclusion body despite extensive optimization of expression conditions; and thus purified under denaturing conditions. This observation is completely consistent with the previous *in vivo* results that FUS is an intrinsically aggregation-prone protein.

Exactly as we previously observed on other aggregation-prone proteins^21–24^ such as TDP43 N-domain^23^ and C-terminal prion-like domain^24^, the purified full-length FUS and its LC domains were also highly soluble and stable in Milli-Q water at pH 4.0, and this allowed us to prepare the protein samples in 1 mM phosphate buffers at pH 5.0 and 6.8 respectively by quickly diluting their stock samples in Milli-Q water to phosphate buffers as we used for studying TDP43 prion-like domain^24^. Figure 1C presents the far-UV CD spectra of the full-length FUS and five dissected fragments at pH 5.0 and 6.8 respectively. The full-length FUS has CD spectra with the maximum negative signals at 200 nm (pH 5.0) and 202 nm (pH 6.8) respectively, which are both lacking of positive signal below 200 nm. The results indicate that FUS at both pH values is not completely random coil but absent of overall tertiary packing^24^. Furthermore, both N- and C-terminal LC domains have CD spectra with the maximum negative signals at ~198 nm at two pH values, and are also lacking of any positive signal below 200 nm, strongly indicating that they are highly disordered in solution. By contrast, the RRM domain has CD spectra very similar at both pH values, which have the similar maximum negative signals at ~214 nm and large positive signals at ~195 nm. The result suggests that RRM is folded and rich in β-sheet at both pH values, completely consistent with it NMR structure previously determined^17^.

We also collected their near-UV CD spectra (Fig. 1D). Both N- and C-terminal LC domains have no significant signal, implying that they are lacking of tertiary packing, consistent with their intrinsically disordered nature. On the other hand, the RRM domain has the maximal negative signal at ~280 nm, indicating that it has tertiary packing typical of a well-folded protein. Interestingly, for the full-length FUS, in addition to the maximal negative signal at ~280 nm characteristic of the folded RRM domain, there is the maximal positive signal at ~308. Furthermore, the near-UV spectrum of the full-length FUS is very different from the simple addition of three spectra of N-, C-terminal LC domains and RRM, implying that in the full-length FUS, three domains might have interactions.

**Figure 2:**
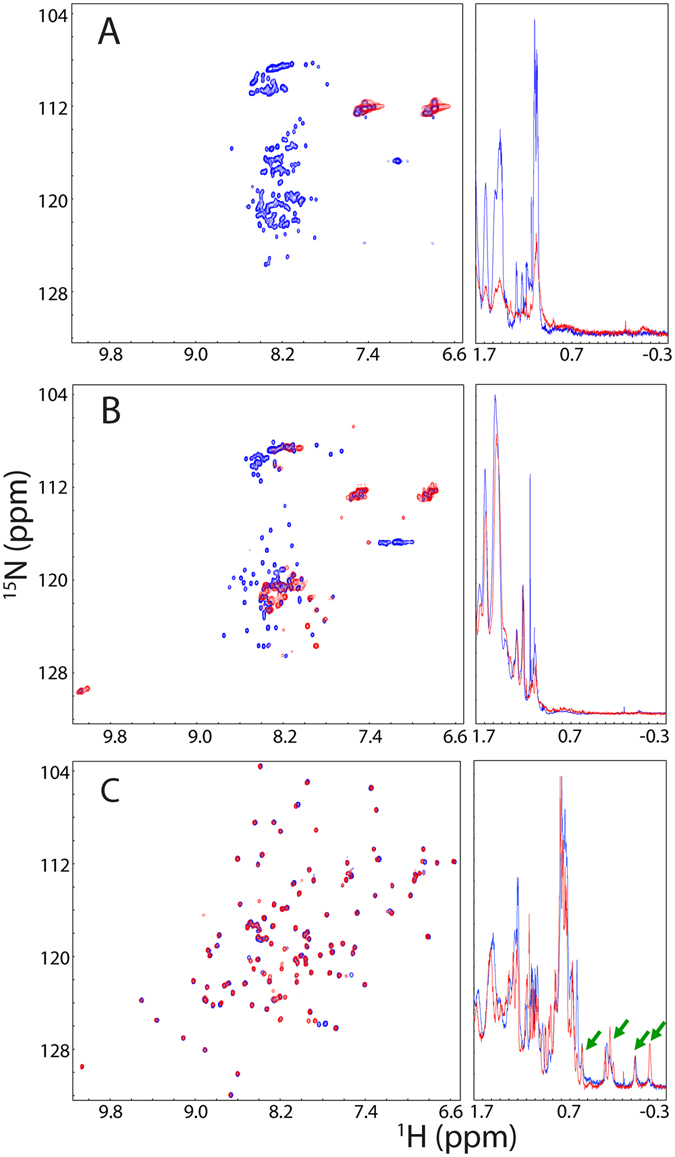
NMR characterization of three dissected domains. Superimposition of two-dimensional (2D) ^1^H-^15^N HSQC and one-dimensional (1D) ^1^H spectra of the N-terminal LC domain (1–267) (**A**); C-terminal LC domain (371–526) (**B**); and RRM (282–371) (**C**), collected at a protein concentration of 40 µM at 25 °C in 1 mM phosphate buffer at pH 5.0 (blue) and pH 6.8 (red). The green arrows are used to indicate up-field peaks manifested in 1D spectra characteristic of a well-folded protein.

We further characterized their solution conformations by one-dimensional (1D) ^1^H and two-dimensional (2D) ^1^H-^15^N NMR HSQC spectra (Fig. 2). Consistent with CD results, both N- and C-terminal LC domains have very narrowly-dispersed HSQC spectra and 1D spectra lacking of any up-field peak at pH 5.0 (Fig. 2A and B), indicating that they are highly disordered. At pH 6.8, the majority of HSQC peaks disappeared for both LC domains, which may be due to the rapid exchange between amide protons and water at neutral pH over the disordered regions, or/and the self-association into large oligomers, or/and the enhancement of µs-ms dynamics. Interestingly, significant intensity reduction and resonance broadening were observed for 1D spectrum of the N-terminal LC domain at pH 6.8, over −0.4–1.8 ppm regions which are resulting from non-exchangeable protons. This implies that at pH 6.8, the N-terminal LC domain undergoes a significant self-assembly as previously reported on the FUS prion-like sequence over 1–165^26–28^. On the other hand, for the C-terminal LC domain, its 1D spectra over −0.4–1.8 ppm are very similar at pH 5.0 and 6.8, implying that the disappearance of its HSQC peaks might be mainly resulting from the rapid exchange between amide protons and water at neutral pH over the disordered regions. By contrast, the RRM domain has well-dispersed HSQC spectra at both pH 5.0 and pH 6.8, with the ^1^H dispersion of 2.2 ppm and ^15^N dispersion of 28.7 ppm; which also has very up-field peaks even with negative chemical shifts in its 1D spectra (Fig. 2C). In particular, most HSQC peaks at two pH values are superimposable, implying that the isolated RRM is folded with its structures highly similar at pH 5.0 and 6.8.

### RRM is perturbed by the presence of the N- and C-terminal LC domains

**Figure 3:**
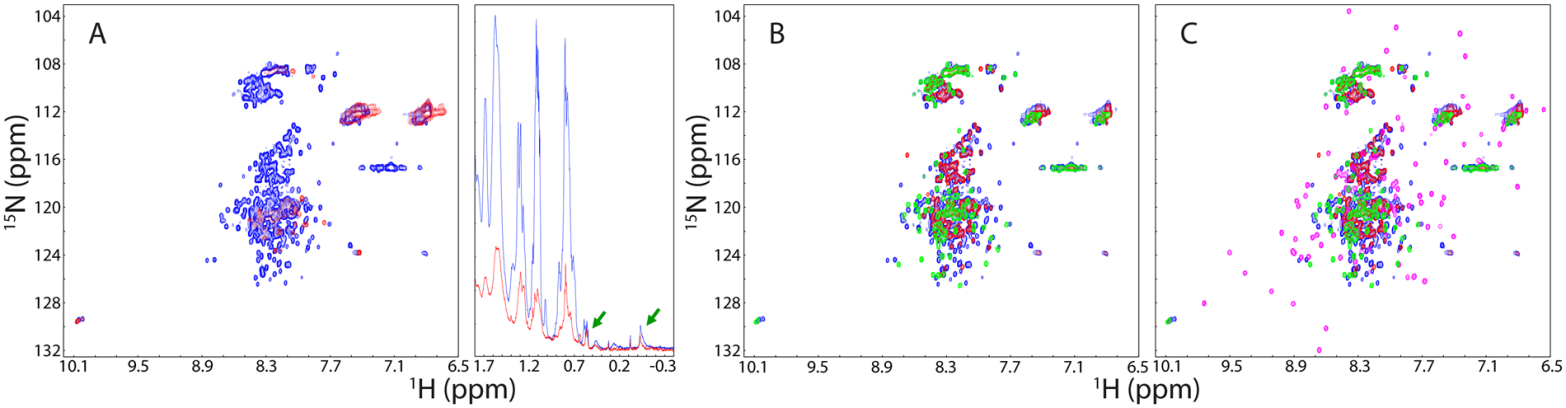
NMR characterization of the full-length FUS. (**A**) Superimposition of 2D NMR HSQC and 1D ^1^H spectra of the full-length FUS protein at a protein concentration of 40 µM in 1 mM phosphate buffer at pH 5.0 (blue) and pH 6.8 (red). The green arrows are used to indicate up-field peaks manifested in 1D spectra characteristic of a well-folded protein. (**B**) Superimposition of 2D NMR HSQC spectra of the full-length FUS protein (blue), N-terminal LC domain (red) and C-terminal LC domain (green) in 1 mM phosphate buffer at pH 5.0. (**C**) Superimposition of 2D NMR HSQC spectra of the full-length FUS protein (blue), N-terminal LC domain (red); RRM (pink); and C-terminal LC domain (green) in 1 mM phosphate buffer at pH 5.0.

Unexpectedly, despite containing the well-folded RRM, even at pH 5.0 the full-length FUS has a narrowly-dispersed HSQC spectrum with only ^1^H dispersion of 1.25 ppm and ^15^N dispersion of 19.2 ppm (Fig. 3A), which has no well-dispersed peaks characteristic of the isolated RRM domain. Nevertheless, despite being broad, there are several very up-field peaks in its 1D spectrum characteristic of a well-folded protein. Furthermore, at pH 6.8, most HSQC peaks disappeared and the 1D peaks became further broad and their intensity reduced. Further superimposition of HSQC spectra at pH 5.0 revealed that the HSQC peaks of the full-length FUS appears to be mainly from those of FUS (1–267) and FUS (371–526) (Fig. 3B), and is completely lacking of any well-dispersed peaks characteristic of the isolated RRM domain (Fig. 3C).

As the N- and C-terminal LC domains are highly disordered and could not manifest very up-field 1D peaks alone, the manifestation of very up-field 1D peaks in 1D spectra of the full-length FUS (Fig. 3A) is resulting from RRM, or/and inter-domain interactions between RRM and LC domains. Consequently, the disappearance of well-dispersed HSQC peaks of RRM in the full-length FUS indicates that the RRM is folded but has inter-domain interactions with LC domains, which might trigger µs-ms dynamics, or/and significantly slow down the rotational tumbling of RRM, thus leading to the significant broadening of the well-dispersed HSQC peaks.

To further understand this phenomenon, we cloned and expressed FUS (1–371) consisting of the N-terminal LC domain and RRM; as well as FUS (267–526) containing the C-terminal LC domain and RRM (Fig. 1A). Noticeably, at pH 6.8 FUS (1–371) has a far-UV CD spectrum with the maximal negative signal at ~204.5 nm and maximal positive signal at ~194 nm, while FUS (267–526) has a far-UV CD spectrum with the maximal negative signal at ~203.8 nm and maximal positive signal at ~192 nm (Fig. 1C). These results indicate that both fragments have tertiary packing to a certain degree, mostly from the folded RRM. Consistent with the far-UV CD spectra, like the isolated RRM, both FUS (1–371) and FUS (267–526) have the maximal negative signals at ~280 nm in their near-UV CD spectra, but FUS (1–371) has the additional maximal positive signal at ~306 nm, similar to what is observed on the full-length FUS. As a consequence, it appears that the maximal negative signals at ~280 are most likely from the folded RRM domain as it is only observed in the full-length FUS and all RRM-containing fragments. On the other hand, the maximal positive signal at ~306 or ~308 nm is likely from the inter-domain interactions between the N-terminal LC domain and RRM because it is only found in the full-length FUS and FUS (1–371) containing both the N-terminal LC domain and RRM.

**Figure 4:**
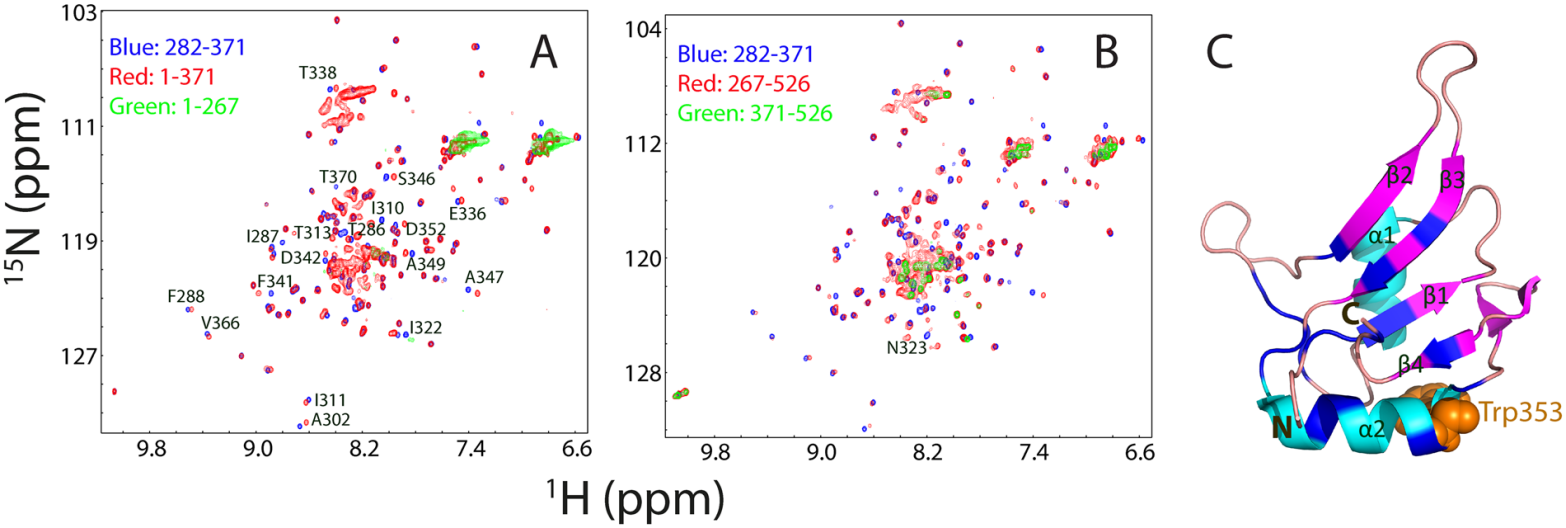
NMR characterization of two RRM-containing fragments. (**A**) Superimposition of HSQC spectra of FUS (1–267), FUS (1–371) and FUS (282–371) in 1 mM phosphate buffer at pH 6.8. The residues with significant shifts of HSQC peaks are labelled for the RRM domain in the isolated state and in the context of FUS (1–371). (**B**) Superimposition of HSQC spectra of FUS (371–526), FUS (267–526) and FUS (282–371) in 1 mM phosphate buffer at pH 6.8. (**C**) NMR structure of the FUS RRM previously determined (PDB ID of 2LCW). The residues with significant shifts of HSQC peaks are colored in blue. The sole Trp353 is displayed as brown spheres.

We also acquired HSQC spectra for FUS (1–371) and FUS (267–526), and subsequently superimposed them respectively to HSQC spectrum of RRM collected at the same conditions (at a protein concentration 0 f 40 µM in 1 mM phosphate buffer at pH 6.8). Interestingly, while many HSQC peaks of RRM are superimposable in the isolated RRM and in FUS (1–371), a portion of HSQC peaks show significant shifts (Fig. 4A). Very intriguingly, a similar shift pattern of HSQC peaks was found in FUS (267–526). This observation implies that some changes in conformations or/and dynamics occur for RRM upon covalently linking to either N-, or C-terminal LC domains. Interestingly, mapping the residues with significant shifts of HSQC peaks back to the RRM structure leads to a very interesting picture that the linkage triggers changes of conformations or/and dynamics of the RRM residues which are distributed over several loop regions, all four β-strands and the first α-helix (Fig. 4C).

To further exclude the possibility that the disappearance of HSQC peaks of RRM in the full-length FUS is due to its purification under denaturing conditions, we also conducted the purification of RRM under denaturing conditions using exactly the same protocol for the full-length FUS, but the obtained RRM protein was also folded at two pH values, with HSQC peaks completely superimposable to those purified under native condition. Furthermore, FUS (1–371) and FUS (267–526) were in fact purified under denaturing conditions but the well-dispersed HSQC peaks of RRM could still be detected. This implies that the disappearance of the well-dispersed HSQC signals of RRM is most likely due to the presence of both N- and C-terminal LC domains. Very amazingly, we have previously observed the same phenomenon on another ALS/FTD-causing protein TDP-43. The well-dispersed HSQC peaks of two well-folded RRM domains also became disappeared in the contexts of the full-length TDP-43 as well as its fragment with the N-terminal domain deleted, which has been characterized to mainly result from µs-ms exchanges between the open and closed conformations mediated by dynamic inter-domain interactions^29^.

### RRM is characteristic of irreversible unfolding

**Figure 5:**
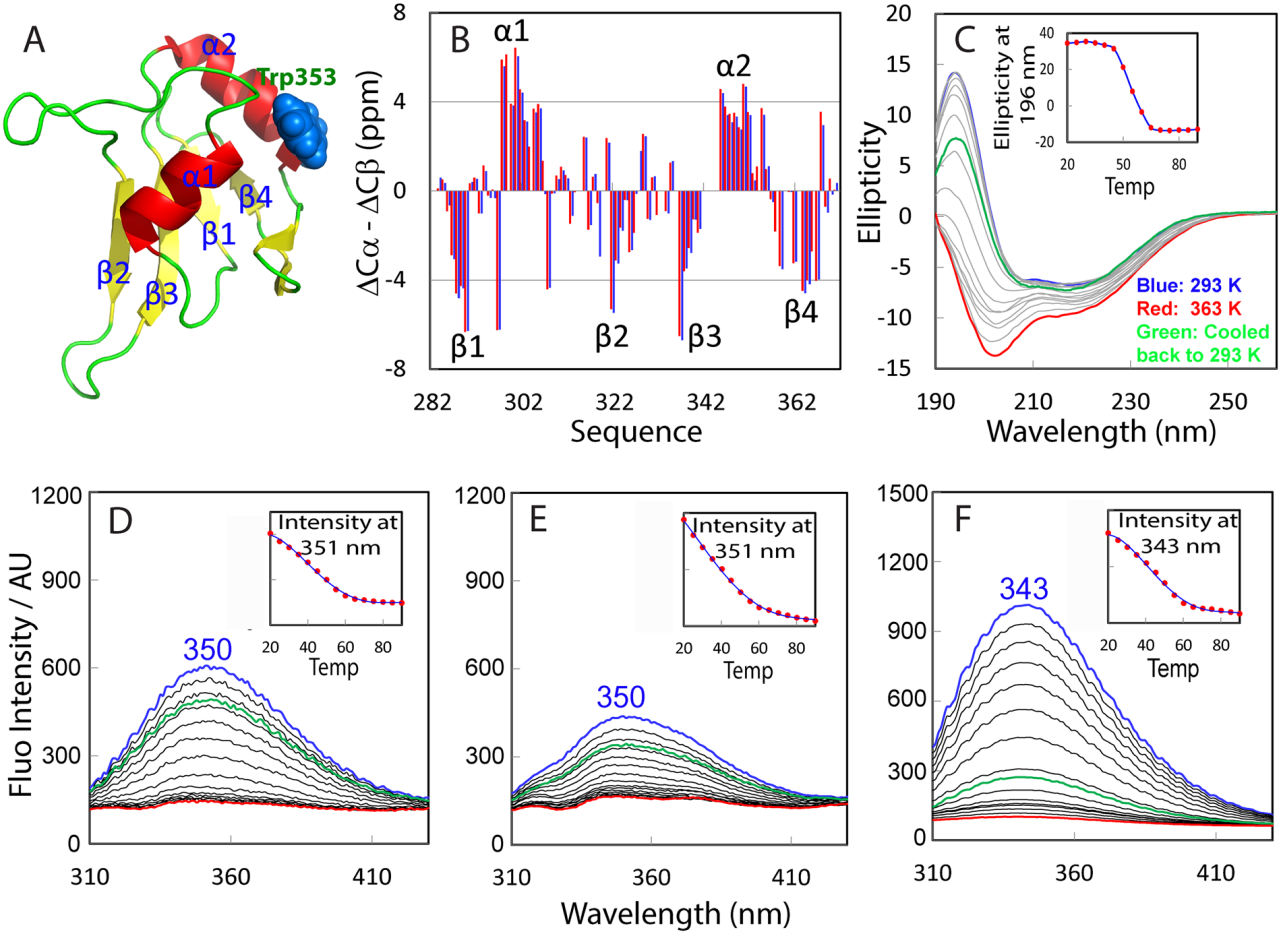
Thermal unfolding of the FUS RRM domain. (**A**) NMR structure of the FUS RRM domain (pdb ID of 2LCW), with secondary structures labelled and Trp353 residue displayed in spheres. (**B**) Residue specific (ΔCα − ΔCβ) chemical shifts of the FUS RRM domain collected at 25 °C in 10 mM phosphate buffer at pH 6.8 (blue) and those previously deposited in BMRB (ID of 17635) (red). (**C**) Far-UV CD spectra of the FUS RRM domain at a concentration of 40 µM in 1 mM phosphate buffer at pH 6.8 with temperatures ranging from 20 to 90 °C. Inlet: The thermal unfolding curve as reported by the changes of ellipiticity at 196 nm. Intrinsic UV fluorescence spectra of the FUS RRM domain at a concentration of 40 µM in 1 mM phosphate buffer at pH 6.8 (**D**); at a concentration of 40 µM in 10 mM phosphate buffer at pH 6.8 (**E**); and at a concentration of 100 µM in 10 mM phosphate buffer at pH 6.8 (**F**) with temperatures ranging from 20 to 90 °C. The fluorescence intensity was reported in arbitrary unit. Inlets: The thermal unfolding curves as reported by the changes of fluorescence intensity at the wavelengths of the emission maxima.

Previously, the FUS RRM domain was determined by NMR to adopt the classical fold as other RRMs with secondary structures of β1–α1–β2–β3–α2–β4 (Figs 4C and 5A). In order to conduct further NMR dynamic studies, we accomplished the sequential assignment of our RRM (282–371) by analyzing a pair of triple resonance spectra HN(CO)CACB and CBCA(CO)NH collected on a ^15^N-/^13^C-double labeled sample. Figure 5B presents the (ΔCα-ΔCβ) chemical shifts, which represent a sensitive indicator of the secondary structures of both folded and disordered proteins^30^. In addition to the N-terminal residue Ser282, as well as Pro320, Pro344, Pro345 and Pro363, only Lys315, Glu336, Asp343 and Ser360 were not assigned due to overlap or undetectable resonance signals. Most (ΔCα-ΔCβ) chemical shifts of the present FUS RRM construct are almost the same as those (BMRB ID of 17635) over the identical region^17^.

We conducted the thermal unfolding of RRM by acquiring a series of far-UV CD spectra at temperatures ranging from 20 to 90 °C (Fig. 5C). By monitoring the change of ellipticity at 196 nm, the melting temperature of the unfolding is estimated to be ~52 °C. However, it seems that even at 90 °C, the RRM domain was not completely unfolded into random coil because the maximum negative signal of the far-UV CD spectrum is at 203 nm although the positive signal below 200 nm is close to 0 (Fig. 5C). An interesting observation is that the CD spectrum of the unfolding sample cooled down back to 20 °C is not superimposable to that of the initial sample at 20 °C. This implies that the unfolding of RRM is irreversible despite containing no Cys residue in its sequence, thus excluding the possibility that the irreversibility is due to the covalent inter-molecular cross-linkage by forming disulfide bridges.

As intrinsic UV fluorescence of Trp residues is very sensitive to the surrounding chemical environments, and FUS RRM only has one Trp353 residue (Fig. 5A), we further used fluorescence spectroscopy to assess the thermal unfolding of FUS RRM at higher protein and buffer concentrations, under which CD spectra will have unacceptably high noises. As reported by intrinsic UV fluorescence of Trp353 (Fig. 5D), under the same condition as used for the above CD study, the intensity gradually reduced with the increase of temperature. Interestingly, the spectrum of the unfolding sample cooled down back to 20 °C is also not superimposable to that of the initial sample at 20 °C. This confirms that the unfolding of RRM is indeed irreversible. Furthermore, in 10 mM phosphate buffer (pH 6.8), RRM at the same protein concentration (40 µM) has intensity slightly reduced but still has the same emission maximum at 350 nm (Fig. 5E), suggesting that the buffer concentrations would not significantly affect the degree of exposure of Trp353. Again under this condition, the thermal unfolding is irreversible. For the RRM sample at a protein concentration of 100 µM in 10 mM phosphate buffer (pH 6.8), the emission maximum shifted from 350 to 343 nm (Fig. 5F). Most interestingly, the spectrum after cooled down back to 20 °C shows much larger difference from the initial one than those at low RRM concentrations, suggesting that the high protein concentration will enhance irreversibility of the unfolding.

**Figure 6:**
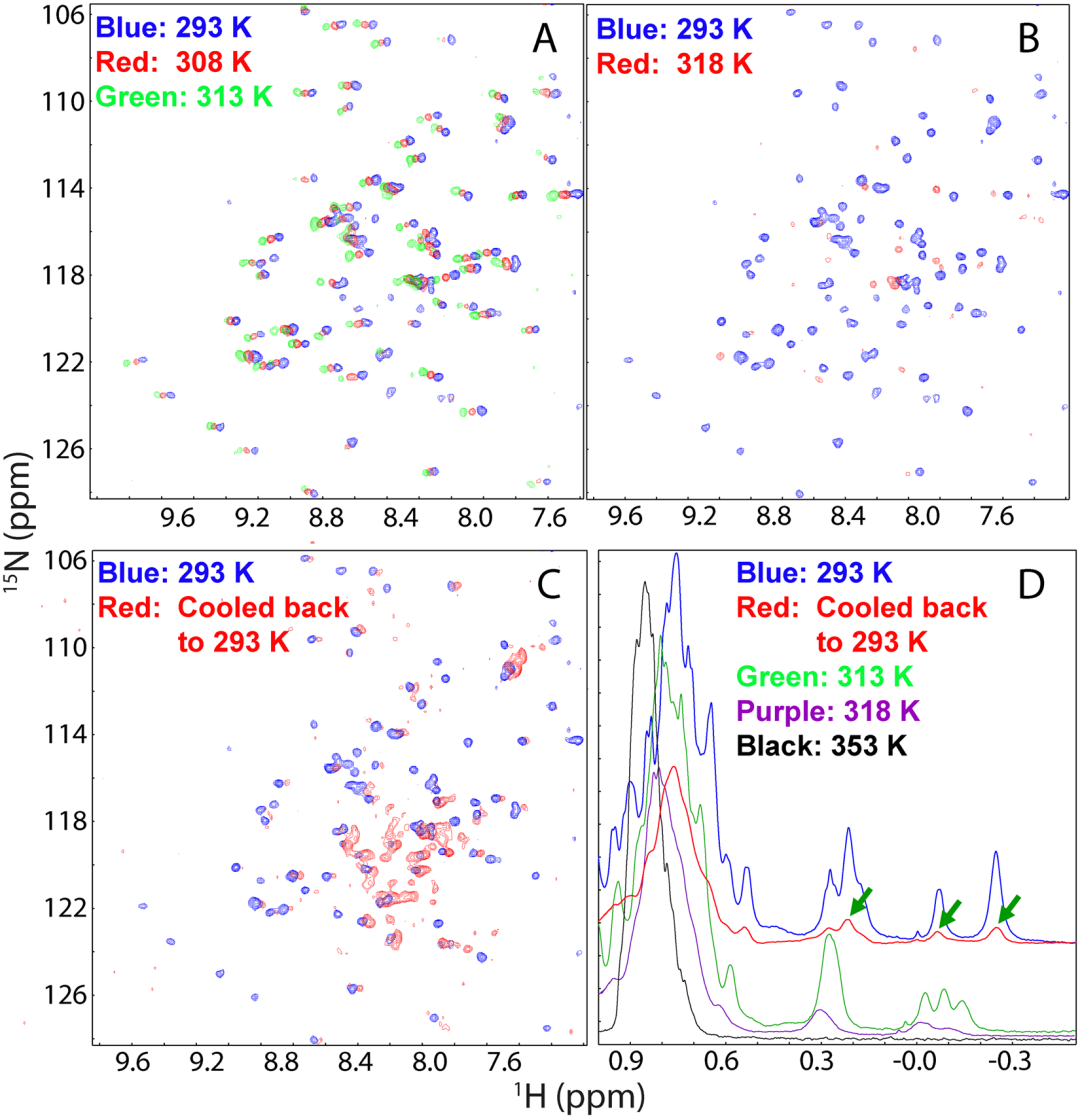
NMR characterization of the thermal unfolding of the FUS RRM domain. (**A**–**C**) ^1^H-^15^N HSQC spectra of the FUS RRM domain at a concentration of 100 µM in 10 mM phosphate buffer at pH 6.8 at different temperatures. (**D**) 1D ^1^H spectra over −0.5–1 ppm at different temperatures. Green arrows are used to indicate several very up-field peaks characteristic of the folded RRM domain.

We also conducted the thermal unfolding of RRM by monitoring HSQC spectra at different temperatures. As shown in Fig. 6A, below 40 °C, most HSQC peaks were still detectable despite having shifts. Once reaching 45 °C, most HSQC peaks disappeared (Fig. 6B), which remained the same even up to 80 °C. Strikingly, the unfolding sample that was cooled down back to 20 °C has a HSQC spectrum different from the initial one, in which most well-dispersed peaks are no longer detectable and the remaining peaks become very broad (Fig. 6C). However, the observations on HSQC peaks are expected to result from the combined effects of enhanced exchanges of amide protons with water at high temperatures, or/and provoked µs-ms conformational exchanges, or/and dynamic self-association. Figure 6D presents 1D proton NMR spectra at different temperatures over −0.5–1 ppm which are from the non-exchangeable methyl protons. As such, the line broadening observed over this region only reflects enhanced µs-ms conformational exchanges, or/and dynamic self-association. At 20 °C, there are very up-field peaks characteristic of the folded RRM domain. At 40 °C, both chemical shifts and shapes of these peaks changed, and in particular, there is a significant line broadening from 40 to 45 °C. Furthermore, all these peaks disappeared at 80 °C, implying a complete elimination of the tertiary packing. Very interestingly, the sample cooled down back to 20 °C still has these up-field peaks with chemical shifts very similar to those of the initial sample at 20 °C. Nevertheless, consistent with the result that the majority of its HSQC peaks are undetectable for the sample cooled down from the thermal unfolding (Fig. 6C), the very up-field peaks also become very broad, implying that the RRM domain refolded from thermal unfolding might already have a similar tertiary packing but undergoes µs-ms conformational exchanges or/and dynamic self-association. Also the NMR sample became gel-like although no visible aggregates formed immediately after the NMR experiments. Together, the unfolding results by CD, fluorescence and NMR spectroscopy reveal that the FUS RRM domain has an irreversible unfolding most probably because the unfolded RRM is prone to the irreversible self-association, thus blocking its complete refolding to the initial structure.

### Backbone dynamics of the RRM domain on ps-ns and µs-ms time scales

**Figure 7:**
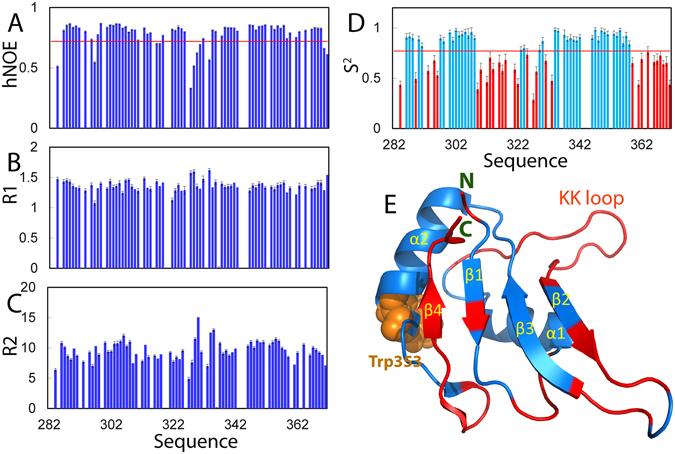
NMR dynamics of the FUS RRM domain. NMR relaxation data of the FUS RRM domain collected at 25 °C at a protein concentration of 400 µM in 10 mM phosphate buffer at pH 6.8: (**A**) hNOE with the average value of 0.72 displayed as red line; (**B**) R1 and (**C**) R2. (**D**) Generalized squared order parameter (S^2^) of the FUS RRM domain. Light blue bars indicate residues with S^2^ > 0.77 while red bars for residues with S^2^ < 0.77 (average value as displayed as red line). (**E**) NMR structure of the FUS RRM domain (pdb ID of 2LCW) with residues having S^2^ < 0.77 colored in red.

We further acquired and analyzed ^15^N NMR backbone relaxation and CPMG-based relaxation dispersion data in order to gain insights into the conformational dynamics of the FUS RRM domain on both ps-ns and µs-ms time scales. ^15^N NMR backbone relaxation data, including the longitudinal relaxation time T1, transverse relaxation time T2, and {^1^H}-^15^N steady-state NOE (hNOE) are sensitive indicators of protein dynamics on the ps-ns timescale^23, 24, 31–35^, and Fig. 7A–C presents the relaxation data for the FUS RRM domain. In addition to 4 Pro and 5 unassigned residues, there are 10 more residues, namely Asp283, Asn285, Leu292, Glu299, Ile311, Gln319, Thr326, Lys332, Asp352 and Lys365, which have significant overlap or/and weak peak intensity. Consequently their relaxation data could not be accurately measured and thus not included here for analysis.

Interestingly, the FUS RRM domain has relatively low hNOE values as compared to other folded proteins such as EphA4 ectodomain even containing several long loops^31^, with an average of 0.72 only, which is very similar to that (0.7) of the ubiquitin-like (UBL) fold adopted by the TDP-43 N-domain existing in equilibrium between the folded and unfolded states exchangeable at ~14 Hz^23^. To gain a quantitative insight, the NMR relaxation data were further analyzed by “Model-free” formalism^31–35^. This analysis generated squared generalized order parameters, S^2^, which reflects the conformational rigidity on ps-ns time scale. S^2^ values range from 0 for high internal motion to 1 for completely restricted motion in a molecular reference frame. The FUS RRM domain has a S^2^ average of 0.77, much smaller than that of EphA4 (0.86)^31^. As shown in Fig. 7D and E, the N- and C-terminal residues, as well as the majority of residues over loops/turns, particularly the unique KK-loop, have S^2^ values smaller than the average. Noticeably, several residues located within β1, β2 and β3 strands also have S^2^ values smaller than the average. In particular, all C-terminal residues from Phe359 including the whole β4 strand also have S^2^ values smaller than the average (Fig. 7E). These results indicate that the FUS RRM domain has a relatively high conformational dynamics on ps-ns time scale.

“Model-free” analysis also yields Rex, which reflect conformational exchanges on µs-ms time scale. Interestingly, the analysis showed that no residue of FUS RRM has Rex, suggesting the global absence of µs-ms dynamics. To independently conform this, we also acquired and analyzed the CPMG-based relaxation dispersion data, and indeed we found no residue with significant relaxation dispersion, thus suggesting that significant µs-ms conformational exchanges are absent for the FUS RRM domain, or/and that the chemical shift differences between exchanging states are too small to be detected by CPMG-based relaxation dispersion experiments^31^. However, as both “Model-free” analysis and CPMG-based relaxation dispersion data detected no µs-ms dynamics, the isolated FUS RRM domain is most likely absent of significant µs-ms conformational exchanges.

### RRM domain spontaneously self-assembles into amyloid fibrils

**Figure 8:**
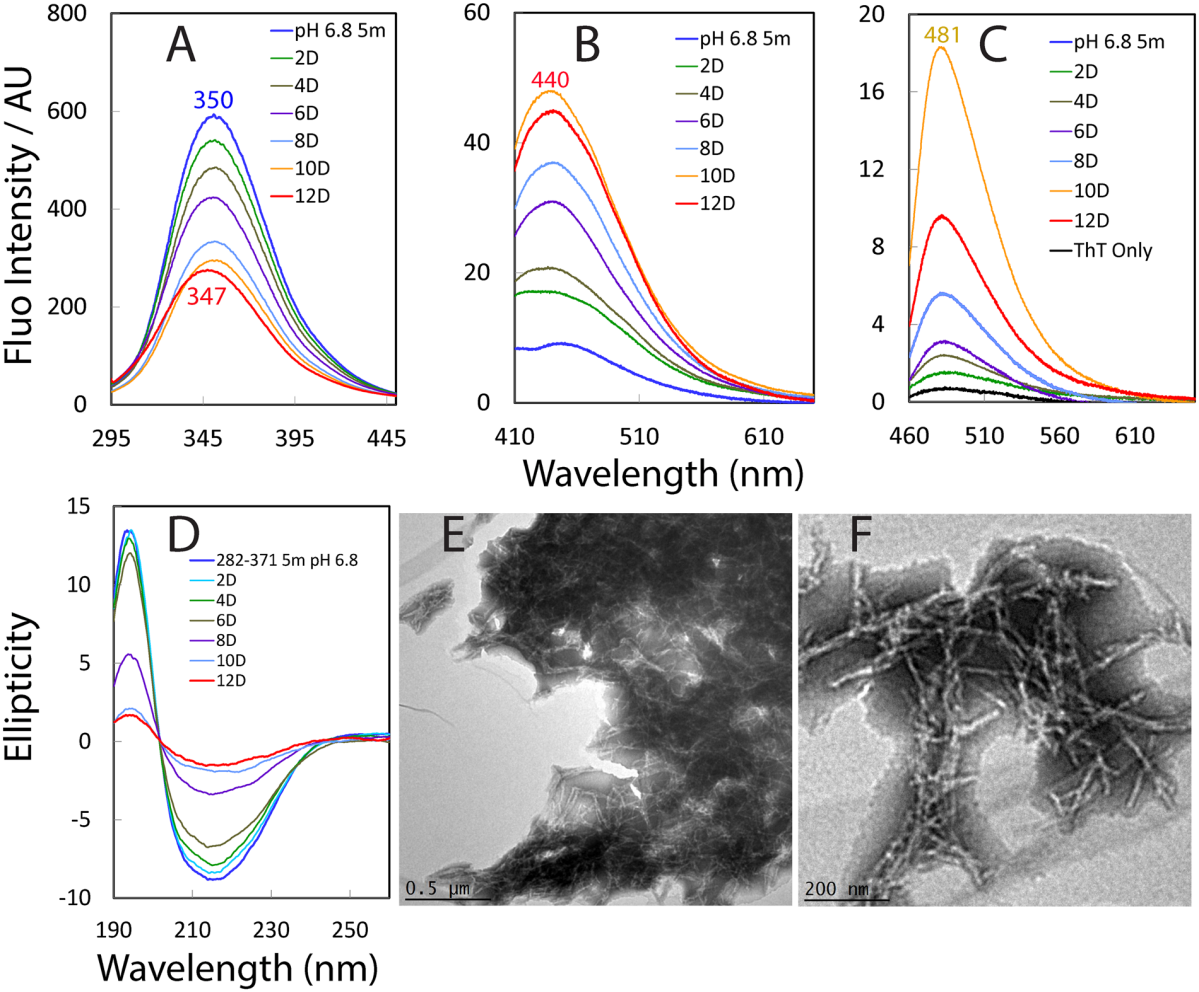
Spontaneous self-assembly of the FUS RRM domain. Emission spectra of the intrinsic UV (**A**); and visible fluorescence (**B**) of the FUS RRM domain at a protein concentration of 40 µM in 1 mM phosphate buffers at pH 6.8 at different time points of the incubation at 25 °C. Wavelengths of the emission maxima are labelled. (**C**) Emission spectra of the ThT-binding induced fluorescence for the FUS RRM domain, which have the typical emission maximum at ~486 nm. (**D**) Far-UV CD spectra of the FUS RRM domain at a protein concentration of 40 µM in 1 mM phosphate buffers at pH 6.8 at different time points of the incubation. EM images of the FUS RRM domain sample at the lower magnification (scale bar of 0.5 μm) (**E**); and higher magnification (scale bar of 200 nm) after 10 days of the incubation.

To assess the role of the RRM domain in the FUS self-assembly, we monitored its self-assembly at 25 °C with a protein concentration of 40 µM in 1 mM phosphate buffer (pH 6.8) by three fluorescence probes and CD spectroscopy as well as EM imaging at different time points up to 12 days (Fig. 8).

Fluorescence spectroscopy is one of the most commonly utilized techniques to characterize the protein self-association^24, 36–39^. Briefly, we monitored the time-lapsed changes of three fluorescence probes, namely the intrinsic UV (Fig. 8A), visible fluorescence (Fig. 8B) and induced fluorescence upon binding to Thioflavin T (ThT) (Fig. 8C). As shown in Fig. 8A, RRM has an initial spectrum of intrinsic UV fluorescence with the emission maximum at ~350 nm. Interestingly, with lapse of time during the incubation, the fluorescence intensity continuously reduced, suggesting that even for the folded RRM, the chemical environment around Trp353 is constantly changing. After 12 days, its emissions maximum shifted to ~347 nm, suggesting that the relatively exposed Trp353 in the initial monomeric state became more and more buried during the incubation, most likely resulting from the self-assembly.

Previously it has been found that β-rich structures could have intrinsic visible fluorescence in both crystal and solution^37, 38^, and in particular this intrinsic visible fluorescence has been demonstrated to gradually develop during the β-rich fibrillar aggregation of amyloid-β (1–40) and (1–42), lysozyme as well as tau^39^. We also found the gradual development of the intrinsic visible fluorescence during the self-assembly of the TDP-43 C-terminal domain (CTD) containing a hydrophobic fragment flanked by two prion-like regions^24^. Most remarkably this intrinsic visible fluorescence has been characterized to have its origin in the formation of special hydrogen bond networks involved in the backbone C=O and N-H atom groups of peptide bonds, which already have electron delocalization to some degree. The formation of the cross-β fibrillar structures with highly aligned hydrogen bonds will further enhance electron delocalization and thus allow low energy electronic transitions required for the manifestation of this intrinsic visible fluorescence^24, 37–39^.

Figure 8B presents the intrinsic visible fluorescence spectra of the FUS RRM domain at different time points. Immediately after initiation of the self-assembly, the FUS RRM domain only has a very weak emission. However, during the incubation the intrinsic visible fluorescence gradually developed with the intensity increased continuously. After 10 days, the fluorescence intensity reached the highest with the emission maximum at ~440 nm, and after the intensity started to reduce slightly. This result clearly indicates that the FUS RRM domain is capable of the self-assembly with a gradual increase of cross-β structures. Furthermore, it is well established that the binding of ThT is a diagnostic probe for formation of the classic amyloid-like structures although the exact molecular details still remain elusive^36^. As seen in Fig. 8C, the intensity of the ThT-binding induced fluorescence also increased gradually and reached the highest after 10 day, independently confirming a significant formation of amyloid fibrils rich in cross-β structures.

Figure 8D presents the far-UV CD spectra of RRM at different time points. With time lapsed, the absolute values of their CD signals became reduced, implying that the FUS RRM self-assembly into large oligomers during the incubation. To visualize the structures formed during the self-assembly, we used electron microscope (EM) to image the incubation samples at 3 days, 6 days and 10 days respectively. Indeed, after 10 days, the FUS RRM domain was found to form fibrillar structure with diameter of 10–20 nm (Fig. 8E and F). Taken together, it can be concluded that the FUS RRM domain is capable of spontaneously self-assembling into amyloid fibrils rich in cross-β structures.

## Discussion

RNA-binding proteins containing the prion-like LC regions such as FUS, TDP-43 and hnRNPA1 have been increasingly identified to be involved in pathological aggregation in ALS and FTD. Recent studies established that FUS can undergo phase separation to self-assemble into physiological and reversible liquid droplet/hydrogel states. On the other hand, many factors such as long incubation times and ALS/FTD-associated mutations have been identified to be sufficient to induce further phase transitions into pathological and irreversible fibrillar states^26–28, 40–43^. However, the high-resolution biophysical mechanism for switching reversibility to irreversibility remains almost unknown although it has been extensively recognized to lie at the heart of ALS and other age-related diseases^5–8, 24, 26–28, 40–51^.

In the present study, we attempted to explore this issue by an integrated use of various biophysical tools. First, as facilitated by our previous discovery^21–24^, we have successfully characterized the solution conformations of the full-length FUS and its five differentially dissected domains. The results revealed that both N- and C-terminal LC domains are intrinsically disordered and only the RRM domain is folded. However, likely due to the interactions of the RRM domains with both N- and C-terminal LC domains, in the full-length FUS the well-dispersed HSQC peaks of the folded RRM domain become undetectable due to significantly provoked µs-ms dynamics, or/and increased rotational tumbling time. This is reminiscent of our previous observation on another ALS-/FTD-causing protein TDP-43^29, 52^. The well-dispersed HSQC peaks of two folded RRM domains also disappeared in the full-length TDP-43 and even its truncated fragment with the N-domain (1–101) deleted, which was characterized to result from µs-ms exchanges between the closed and open conformations mediated by dynamic inter-domain interactions coordinated by intrinsically-disordered prion-like domain^29^. Despite being very challenging, in the future it would be important to identify the exact regions involved in inter-domain interactions of FUS. It is also of fundamental interest to examine whether other RNA-binding proteins containing RRM and LC regions also share this feature. If yes, what is their functional relevance/advantage?

We further conducted extensive characterizations on the thermal unfolding and NMR dynamics on the FUS RRM domain. The results decipher that it is characteristic of an irreversible unfolding even at a low protein concentration. Most likely, this property is likely resulting from the association-prone capacity for the FUS RRM domain upon being denatured. Quantitative analysis of NMR ^15^N backbone relaxation data decodes that it also has a relatively high backbone dynamics on ps-ns time scale. More precisely, in addition to terminal and loop residues, residues even within four β-strands also have high conformational dynamics (Fig. 7E). This result implies that the barrier to separate the folded and unfolded states of the FUS RRM domain might be relatively low and these states are exchanging on a time scale even faster than µs-ms, as CPMG-based relaxation dispersion experiments detected no µs-ms conformational exchange. Amazingly, however, upon interacting with both N- and C-terminal LC domains in the full-length FUS, the RRM domain appears to acquire global µs-ms dynamics, or/and have a much longer rotational tumbling time, thus leading to the disappearance of its well-dispersed HSQC peaks.

Another interesting finding is that the FUS RRM domain can spontaneously assemble into amyloid fibrils. Previously, it has been extensively revealed that the FUS prion-like domain over residues 1–165 drives the FUS self-assembly into reversible and physiological droplets/hydrogels^26–28^. Here, we have identified the FUS RRM domain to be also capable of the self-assembly in addition to the well-established prion-like domain. The feature of the FUS RRM domain to unfold irreversibly implies that it may play an important role in switching the reversible and physiological self-assembly into irreversible and pathological states.

Our current study reveals that like TDP-43^23, 24, 29, 52–55^, the dynamics and self-assembly of FUS are also modulated by more than one domain/region, as well as by their interactions. As a consequence, various perturbations including genetic, pathological and environmental factors to one domain may be relayed to other domains to solidifying the reversible and physiological FUS assembly, thus leading to ALS/FTD by “loss-of-function” or/and “gain-of-function” mechanisms. Indeed, we recently found that by differentially disrupting the inter-domain interactions, ALS-causing cleavages of TDP-43 to remove its N-terminal regions are in fact able to unlock its CTD in a stepwise manner for enhanced aggregation and toxicity of the pathological C-terminal fragments^52^. Furthermore, Asp169 was also shown to be involved in the inter-domain interactions^52^, and consequently D169G, the only ALS-causing mutation within RRMs might also gain enhanced aggregation and toxicity by altering the inter-domain interactions. In this context, our current study may explain that while no ALS-/FTD-causing mutation has been found on the RRM domain of FUS so far, unexpectedly the previous study identified its RRM domain to be essential for manifesting cytotoxicity of FUS *in vivo* ^20^.

## Methods

### Preparation of recombinant proteins

The DNA fragments encoding the full-length FUS and its five dissected domains (Fig. 1A) were amplified by PCR reactions from FUS cDNA and subsequently cloned into a modified vector pET28a as we previously used for the TDP-43 prion-like domain^24^. The expression vectors were subsequently transformed into and overexpressed in *Escherichia coli* BL21 (DE3) cells (Novagen). The recombinant proteins of the full-length FUS, FUS (1–267), FUS (1–371), FUS (267–526) and FUS (371–526) were found only in inclusion body while RRM was highly soluble in supernatant. As a result, for the full-length FUS and its four aggregation-prone fragments, the pellets were first dissolved in a phosphate buffer (pH 8.5) containing 8 M urea and subsequently purified by a Ni^2+^-affinity column (Novagen) under denaturing conditions in the presence of 8 M urea. The fractions containing the recombinant proteins were acidified by adding 10% acetic acid and subsequently purified by reverse-phase (RP) HPLC on a C4 column eluted by water-acetonitrile solvent system. The HPLC elution containing pure recombinant proteins were lyophilized.

For RRM, the recombinant proteins were purified by a Ni^2+^-affinity column (Novagen) under native condition, followed by a further purification by FPLC on a gel-filtration column. However, to assess whether the purification under denaturing conditions would affect the folding of RRM, we also conducted purification under denaturing conditions using the same protocol as for the full-length FUS and its four aggregation-prone fragments.

The generation of the isotope-labelled proteins for NMR studies followed a similar procedure except that the bacteria were grown in M9 medium with the addition of (^15^NH_4_)_2_SO_4_ for ^15^N labeling and (^15^NH_4_)_2_SO_4_/[^13^C]-glucose for double labelling^23, 24, 29, 31^. The purity of the recombinant proteins was checked by SDS–PAGE gels and their molecular weights were verified by a Voyager STR matrix-assisted laser desorption ionization time-of-flight-mass spectrometer (Applied Biosystems). The concentration of protein samples was determined by the UV spectroscopic method in the presence of 8 M urea. Briefly, under the denaturing condition, the extinct coefficient at 280 nm of a protein can be calculated by adding up the contribution of Trp, Tyr and Cys residues^56^.

### CD and NMR experiments

All circular dichroism (CD) experiments were performed on a Jasco J-1500 spectropolarimeter equipped with a thermal controller as previously described^23, 24^. Far-UV CD spectra were collected in 1-mm curvet while near-UV CD spectra were in 10-mm curvet because much high protein concentrations are needed to obtain high-quality near-UV spectra. Data from five independent scans were added and averaged. CD samples of the full-length FUS and its four aggregation-prone fragments were prepared by diluting the concentrated samples (~200 µM) dissolved in Milli-Q water (pH 4.0) into 1 mM phosphate buffers to obtain samples at a protein concentration of 40 µM with final pH at pH 5.0 and 6.8 respectively.

All NMR experiments were acquired on an 800 MHz Bruker Avance spectrometer equipped with pulse field gradient units as described previously^23, 24, 29, 31, 52^. For characterizing the solution structure of the FUS RRM domain, a pair of triple-resonance experiments HNCACB, CBCA(CO)NH were collected for the sequential assignment on a ^15^N-/^13^C-double labelled sample of 400 µM. NMR data were processed with NMRPipe^57^ and analyzed with NMRView^58^.

### Fluorescence spectral measurements

For monitoring the self-assembly of the FUS RRM domain, fluorescence spectra were measured at 25 °C with a RF-5301 PC spectrophotometer (Shimadzu, Japan) as previously described^24^ at different time points of the incubations at 25 °C at a protein concentration of 40 µM in 1 mM phosphate buffer (pH 6.8). The rectangular fluorescence quartz cuvette has the pathlength dimension of 10 × 10 mm and the general settings are: PMT at low sensitivity and scan speed of medium speed (200 nm/min). For the intrinsic UV fluorescence, the emission spectra were measured with the excitation wavelength at 280 nm and slit widths: excitation at 5 nm and emission at 10 nm. For the intrinsic visible fluorescence, the emission spectra were measured with the excitation wavelength at 375 nm and slit widths: excitation at 20 nm and emission at 10 nm.

For Thioflavin-T (ThT) binding assay, a 2 mM ThT stock solution was prepared by dissolving ThT in milli-Q water and filtered through a 0.22 μm Millipore filter. The fresh working solution was prepared by diluting the stock solution into 1 mM phospate buffer (pH 6.8) to reach a final ThT concentration of 50 μM. A 10 μL aliquot of each incubation solution, or 10 μL aliquot of the incubation buffer (1 mM phosphate at pH 6.8) as the control, was mixed with 130 μL of the ThT working solution in the dark for 10 mins. The fluorescence emission spectra were acquired for three repeats with the excitation wavelength at 442 nm and slit widths: excitation at 5 nm and emission at 10 nm^24^.

### Protein dynamics on ps-ns time scale as studied by NMR spectroscopy

^15^N backbone T1 and T1ρ relaxation times and {^1^H}-^15^N steady state NOE intensities were collected on the ^15^N-labeled RRM domain at 25 °C at a concentration of 400 µM in 10 mM phosphate buffer at pH 6.8 on an Avance 800 MHz Bruker spectrometer with both an actively shielded cryoprobe and pulse field gradient units^23, 24, 29, 31^. Relaxation time T1 was determined by collecting 7 points with delays of 10, 160, 400, 500, 640, 800 and 1000 ms using a recycle delay of 1 s, with a repeat at 400 ms. Relaxation time T1ρ was measured by collecting 7 points with delays of 1, 12, 30, 40, 50, 80, 100 ms. {^1^H}-^15^N steady-state NOEs were obtained by recording spectra with and without ^1^H presaturation, a duration of 3 s and a relaxation delay of 6 s at 800 MHz.

### Model-free analysis

NMR relaxation data were analyzed by “Model-Free” formalism with protein dynamics software DYNAMICS^31–35^. Briefly, relaxation of protonated heteronuclei is dominated by the dipolar interaction with the directly attached ^1^H spin and by the chemical shift anisotropy mechanism. Relaxation parameters are given by:

1 $${R}_{1}={d}^{2}/4[J({\omega }_{H}-{\omega }_{X})+3J({\omega }_{X})+6J({\omega }_{H}+{\omega }_{X})]+{c}^{2}J({\omega }_{X})$$

2 $$\begin{array}{rcl}{R}_{2} & = & {d}^{2}/8[4J(0)+J({\omega }_{H}-{\omega }_{X})+3J({\omega }_{X})+6J({\omega }_{H})+6J({\omega }_{H}+{\omega }_{X})]\\  &  & +({c}^{2}/6)[4J(0)+3J({\omega }_{X})]+{R}_{ex}\end{array}$$

3 $$NOE=1+({d}^{2}/4{R}_{1})({\gamma }_{X}/{\gamma }_{H})[6J({\omega }_{H}+{\omega }_{X})-J({\omega }_{H}-{\omega }_{X})]$$

In which, $d={\mu }_{0}{\gamma }_{X}{\gamma }_{H}\langle {\gamma }_{XH}^{-3}\rangle /8{\pi }^{2},c={\omega }_{X}{\rm{\Delta }}\sigma /\sqrt{3}$, *μ*_0_ is the permeability of free space; *h* is Planck’s constant; *γ*_*X*_, *γ*_*H*_ are the gyromagnetic ratios of ^1^H and the X spin (X = ^13^C or ^15^N) respectively; *γ*_*XH*_ is the X-H bond length; *ω*_*H*_ and *ω*_*X*_ are the Larmor frequencies of ^1^H and X spins, respectively; and Δ*σ* is the chemical shift anisotropy of the X spin.

The Model-Free formalism, as previously established^32^ and further extended^35^, determines the amplitudes and time scales of the intramolecular motions by modeling the spectral density function, *J*(*ω*), as

4 $$J(\omega )=\frac{2}{5}[\frac{{S}^{2}{\tau }_{m}}{1+{(\omega {\tau }_{m})}^{2}}+\frac{({{S}_{f}}^{2}-{S}^{2})\tau }{1+{(\omega \tau )}^{2}}]=\frac{2}{5}{{S}_{f}}^{2}[\frac{{{S}_{s}}^{2}\tau m}{1+{(\omega {\tau }_{m})}^{2}}+\frac{(1-{{S}_{s}}^{2})\tau }{1+{(\omega \tau )}^{2}}]$$

In which, $\tau ={\tau }_{s}{\tau }_{m}/({\tau }_{s}+{\tau }_{m})$, *τ*_*m*_ is the isotropic rotational correlation time of the molecule, *τ*_*s*_ is the effective correlation time for internal motions, *S* ^2^ = *S*_*f*_^2^ *S*_*s*_^2^ is the square of the generalized order parameter characterizing the amplitude of the internal motions, and *S*_*f*_^2^ and *S*_*s*_^2^ are the squares of the order parameters for the internal motions on the fast and slow time scales, respectively.

In order to allow for diverse protein dynamics, several forms of the spectral density function, based on various models of the local motion, were utilized, which include the original Lipari-Szabo approach, assuming fast local motion characterized by the parameters *S* ^2^ and *τ*_*loc*_; extended model-free treatment, including both fast (*S*_*fast*_^2^, *τ*_*fast*_) and slow (*S*_*slow*_^2^, *τ*_*slow*_) reorientations for the NH bond (*τ*_*fast*_ << *τ*_*slow*_ < *τ*_*c*_); and could also allow for slow, milli- to microsecond dynamics resulting in a conformational exchange contribution, *R*_*ex*_, to the linewidth. In DYNAMICS, there are eight models for local motions and each residue is fitted with different models. Subsequently goodness of fit will be checked and the best-fitted model will be selected^34, 59^.

The relaxation data of the FUS RRM domain were analyzed with the previously published NMR structure (pdb ID of 2LCW)^17^ by isotropic, axially-symmetric and fully anisotropic models for the overall motion and the results were tested and then compared. According to the illustration of ROTDIF, axially-symmetric model was finally selected because of smallest Ch^2^/df value^34, 59^, which has τc of 6.9 ns; Dx = Dy = 2.56 ± 0.15; Dz = 3.04 ± 0.46 (10^7^ S^−1^) and alpha = 108 ± 24; beta = 48 ± 45 degree.

### Protein dynamics on µs-ms time scale as studied by NMR spectroscopy

^15^N transverse relaxation dispersion experiments were acquired on the ^15^N-labeled RRM domain on a Bruker Avance 800 spectrometer. A constant time delay (*T*_CP_ = 50 ms) was used with a series of CPMG frequencies, ranging from 40 Hz, 80 Hz, 120 Hz (x2), 160 Hz, 200 Hz, 240 Hz, 320 Hz, 400 Hz, 480 Hz, 560 Hz, 640 Hz, 720 Hz, 800 Hz, and 960 Hz (x2 indicates repetition). A reference spectrum without the CPMG block was acquired to calculate the effective transverse relaxation rate by the following equation:

5 $${R}_{2}^{eff}=-\mathrm{ln}(I({\nu }_{CPMG})/{I}_{0})/{T}_{CP}$$

where I(ν_CPMG_) is the peak intensity on the difference CPMG frequency, I_0_ is the peak intensity in the reference spectra^31^.

### Electron microscopy imaging

Incubation samples of the FUS RRM domain at 25 °C at a protein concentration of 40 µM were imaged at 3, 6 and 10 days of the incubation in 1 mM phosphate buffer (pH 6.8), by a TEM microscope (Jeol Jem 2010f Hrtem, Japan) operating at an accelerating voltage of 200 kV as previously described^24^.

Briefly, for EM imaging, a 5 µl aliquot of the incubation or aggregate solutions was placed onto the Cu grids (coated with carbon film; 150 mesh; 3 mm in diameter) and negatively stained with 5 μl of 2% neutral, phosphotungstic acid (PTA). This aliquot was allowed to settle on Cu grid for 30 sec before the excess fluid was drained away. The Cu grid was later air-dried for another 15 mins before being imaged.
